# Effect of Laser Acupuncture on Anthropometric Measurements and Appetite Sensations in Obese Subjects

**DOI:** 10.1155/2016/9365326

**Published:** 2016-03-09

**Authors:** Chi-Chuan Tseng, Alan Tseng, Jason Tseng, Chia-Hao Chang

**Affiliations:** ^1^Division of Chinese Medicine, Chang Gung Memorial Hospital, Chiayi 61363, Taiwan; ^2^School of Traditional Chinese Medicine, Chang Gung University, Taoyuan 33302, Taiwan; ^3^Department of Medical Biophysics, University of Toronto, Toronto, ON, Canada M5G 1L7; ^4^Faculty of Arts and Science, University of Toronto, Toronto, ON, Canada M5S 3G3; ^5^Chronic Diseases and Health Promotion Research Center, Chang Gung University of Science and Technology, Chiayi Campus, Chiayi 61363, Taiwan; ^6^Department of Nursing, Chang Gung University of Science and Technology, Chiayi Campus, Chiayi 61363, Taiwan

## Abstract

*Purpose*. A patient-assessor-blinded, randomized, sham-controlled crossover trial was performed to investigate the effectiveness of laser acupuncture on anthropometric measurements and appetite sensation in obese subjects.* Methods*. Fifty-two obese subjects were randomly assigned to either the laser acupuncture group or the sham laser acupuncture group. Subjects within each group received the relevant treatment three times a week for 8 weeks. After a two-week washout period, the subjects then received the treatment of the opposite group for another 8 weeks. BMI, body fat percentage, waist-to-hip ratio (WHR), waist circumference, hip circumference, and appetite sensations were measured before and after 8 weeks of treatment.* Results*. BMI, body fat percentage, WHR, waist circumference, and hip circumference decreased significantly (*p* < 0.05) in the laser acupuncture group compared to baseline but there was no decrease in those variables in the sham laser acupuncture group. Laser acupuncture significantly improved scores on the fullness, hunger, satiety, desire to eat, and overall well-being relative to the baseline (*p* < 0.05).* Conclusions*. Laser acupuncture is well tolerated and improves anthropometric measurements and appetite sensations in obese subjects.

## 1. Introduction

It is estimated that over 10% of the world's adult population suffers from obesity [1]. Obesity is a detriment to quality of life and places emotion and financial burden on the individual, their families, and society [2]. Subjects also have an increased risk of associated conditions, such as coronary heart disease, type II diabetes, stroke, osteoarthritis, and certain cancers [3]. Dietary modification, lifestyle interventions, pharmacological interventions, and bariatric surgery are treatment choices for obesity, but more safe and effective treatment options are in high demand.

Acupuncture treatment has been increasing in popularity but systematic reviews on its effectiveness of weight management intervention are inconclusive [4, 5]. The validity of using minimal acupuncture or placebo needle as an inert placebo control has been questioned [6]. While acupuncture is very low risk when properly performed by well-trained acupuncturists, there are some adverse events resulting from acupuncture which are also a concern [7].

Laser acupuncture (LA) is an intervention that stimulates traditional acupoints using low-level laser therapy (LLLT) [8]. Compared to traditional manual acupuncture, LA has multiple advantages, including ease of application, dose measurement precision, painlessness, and noninvasiveness. It is also quick, safe, and inexpensive and carries no risk of infection [9, 10]. Double blind randomized controlled studies are also much easier to perform due to the lack of local sensation when performing LA [11].

Clinical research on the efficacy of LA has increased over the last 30 years [12, 13]. Previous studies have shown that LA has promising effects on obesity [14, 15]. LLLT as an approach for body contouring and spot fat reduction has also been reported [16, 17]. But the absence of placebo control and lack of sufficient comparative parameters have been questioned [18]. The aim of this study was to investigate the efficacy of LA with gallium aluminum arsenide (GaAlAs) laser irradiation on weight loss, fat reduction, body contouring, and appetite sensations in obese subjects.

## 2. Materials and Methods

### 2.1. Study Design

**Figure 1:**
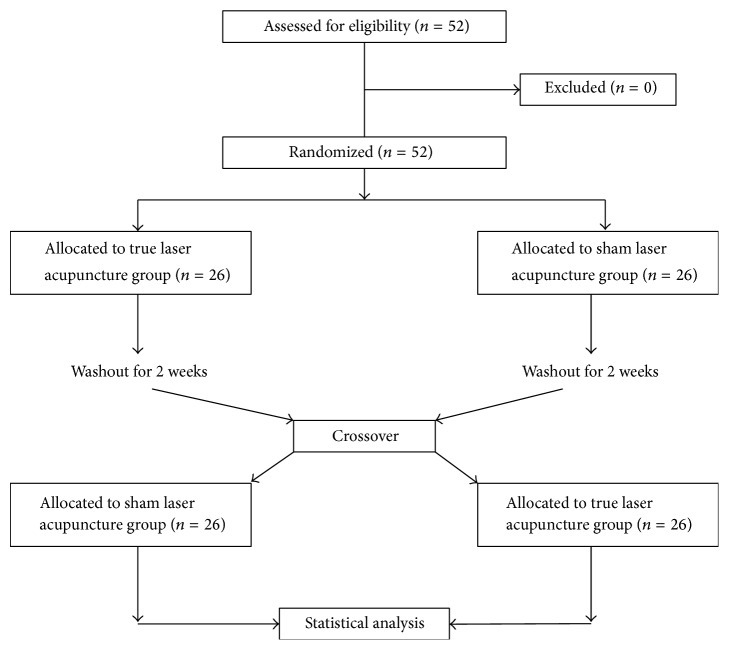
Study flowchart of the laser acupuncture randomized, sham-controlled crossover trial for obesity.

We conducted a patient-assessor-blinded, randomized, sham-controlled crossover trial to investigate the efficacy of laser acupuncture for obesity. Participants were recruited from the community by placing advertisements on websites and around the hospital. Interested subjects were initially screened over the phone to ensure subjects fitted for participation based on inclusion and exclusion criteria. The study was performed at the Chiayi Chang Gung Memorial Hospital. Fifty-two obese subjects were recruited through advertisement. The participants were randomized into a LA treatment group and a sham LA control group. Subjects within each group received the relevant treatment three times a week for 8 weeks. After a two-week washout period, the subjects then received the treatment of the opposite group for another 8 weeks. The study design is depicted in Figure 1.

### 2.2. Randomized Allocation and Blinding

After initial assessments, subjects that met the inclusion criteria and passed the exclusion criteria were randomly assigned to one of two groups with a 1 : 1 allocation ratio according to a computer-generated randomization list. The group designation for each subject was concealed in sequentially numbered sealed opaque envelopes that were only opened after the subject completed baseline clinical assessments. The random group allocation was concealed from the physicians, subjects, and the evaluators.

### 2.3. Participants

The inclusion criteria for the subjects in the study were to be above 20 years of age and have a BMI over 25 kg/m^2^. BMI cutoffs were adopted from a proposed classification of weight by BMI in adult Asians, including the obese I (BMI: 25–29.9 kg/m^2^) and obese II (BMI ≥ 30 kg/m^2^) categories [19]. Participants were excluded from the study if one or more of the following criteria were fulfilled: a history of cardiovascular disease, diabetes, endocrine abnormalities, renal disease, contagious skin condition, epilepsy, tumors, or mental disorders; pregnancy; photosensitivity reactions to laser treatment; use of a pacemaker; use of medications known to affect one's weight up to one month in advance; or unwillingness to comply with the study protocol.

### 2.4. Interventions

Laser acupuncture was applied by a GaAlAs semiconductor diode laser phototherapy device (Model: T-816-3E2-808) developed by Transverse Industries Co., Ltd., Taiwan, with a wavelength of 808 nm. Maximum power output was 150 mW in continuous wave mode at a power density of 0.417 W/cm^2^. The 4 J/cm^2^ energy density laser was applied for 10 seconds to each of the selected acupoints. This is the recommended dosage for LLLT as documented by the World Association of Laser Therapy [20, 21]. The laser handheld device (laser beam spot size ≦36 mm^2^) was applied directly and perpendicularly. To avoid scattering the beam, slight contact was made with the skin surface. Subjects reported no other sensations besides that of the light touch of the laser probe grazing the skin.

Acupoints were chosen based on traditional Chinese medicine (TCM) theory for the obese [22]. The treatment principles were invigorating qi, eliminating spleen dampness, and nourishing the kidney [23, 24]. The acupoints were ST25 (Tianshu), ST36 (Zusanli), ST40 (Fenglong), ST44 (Neiting), LI4 (Hegu), LI11 (Quchi), SP6 (Sanyinjiao), and PC6 (Neiguan) (Table 1). Acupoints, application duration, and total treatment count were identical between the two groups, but subjects in the sham LA group underwent placebo LA treatment under which the laser had no power output. The same physician performed all laser applications.

### 2.5. Outcome Measurements

The primary outcome measurement was the change in BMI (kg/m^2^) from the baseline. Secondary outcome measurements include waist circumference, hip circumference, WHR, body fat percentage, and appetite sensations. Waist circumference was measured using a stretch-resistant tape at the midpoint between the lower margin of the least palpable rib and the top of the iliac crest. Hip circumference was measured at the widest portion of the buttocks with the tape parallel to the floor [25]. Bioelectrical impedance was used to measure body fat percentage.

A 10 cm Visual Analogue Scale (VAS; 0 representing the most negative rating and 10 representing the most positive rating) was used to evaluate change in appetite sensations and overall symptoms. Subjects were asked to mark their level of fullness, hunger, satiety, desire to eat, and overall well-being on the VAS. Subjects were prohibited from accessing past VAS records in subsequent sessions. Measurements of appetite sensations as expressed by ratings on the VAS have been validated extensively for use in appetite research [26]. These clinical assessments were performed before and after each 8-week period.

There were two evaluators who independently evaluated posttreatment and pretreatment parameters. Before the treatment, clinical assessment parameters were evaluated by an evaluator. Posttreatment assessments were evaluated by a different evaluator. Since each set of assessment parameters was only evaluated once, there can be no evaluation differences among evaluators. The physician applying the treatment was not involved with the parameter assessment. The subjects, evaluators, and statistician performing the data analyses were blinded to the treatment allocation throughout the study.

### 2.6. Statistical Analysis

Statistical analyses were performed using the R software version 3.2.2 [27]. Means and standard deviations of the clinical indices were calculated. The differences of evaluation scores before and after the treatments were analyzed with paired *t*-tests. The changes in the sham and true laser groups were compared with additional paired *t*-tests. The *p* values were adjusted for multiple comparisons using the Benjamini and Hochberg method [28] to control for the false discovery rate (FDR). For all the analyses, a value of *p* < 0.05 was regarded as statistically significant. The null hypothesis we used was Δsham = Δtreatment, where Δsham = (measurement at 8 weeks after sham treatment − baseline) and Δtreatment = (measurement at 8 weeks after experimental treatment − baseline). To decrease the subject dropout rate, the trial was designed to only last eight weeks. Assuming a dropout rate of 10%, the desired sample size for this pilot study is 52 subjects, with 26 in each group. Based on the a priori calculation in G ^*∗*^Power 3.1.3, it was determined that a minimum total sample size of 46 was needed in a repeated-measures-between-factors design to show significant results (effect size = 0.25; *α* = 0.05; 1 − *β* = 0.8; number of measurements = 8; correlation among repeated measures = 0.2) [29].

### 2.7. Adverse Events

Subjects were asked to report any adverse events they experienced. The evaluator interviewed the subjects to confirm the validity of the adverse event. If these events occurred during treatment, the physician immediately stopped the procedure and treated the adverse event. The evaluator filed a report detailing the seriousness of the event, event onset date, relationship between the event and the treatment, possible causes of the event other than the trial itself, and other relevant data. The ethics committee decided whether or not to modify the study protocol or remove the subject from the trial.

## 3. Results

A total of 52 obese subjects, mean ± SD age 38.8 ± 11.7 years, mean body mass index 31.1 ± 5.3 kg/m^2^, mean systolic blood pressure 124.5 ± 9.8 mmHg, and mean diastolic blood pressure 75.3 ± 5.1 mmHg, were recruited in the study. There were no dropouts and no adverse reactions were reported. Baseline demographic and anthropometric characteristics of the participants are shown in Table 2.

**Figure 2:**
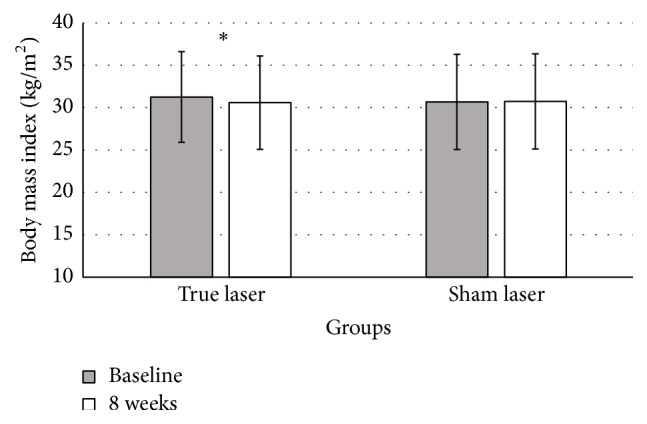
Body mass index before and after 8 weeks of the true LA and sham LA treatments (*n* = 52). One measurement was performed per patient per intervention group per time point. Bars indicate mean of BMI across all patients in each group. Error bars indicate standard deviation. *∗* indicates a significant difference in the measurement at 8 weeks compared with baseline (*p* < 0.05, paired *t*-test).

**Figure 3:**
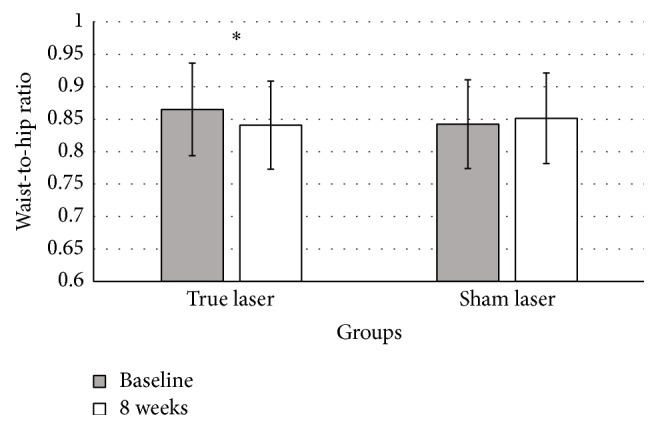
Waist-to-hip ratio before and after 8 weeks of the true LA and sham LA treatments (*n* = 52). Measurements were conducted at the same time as in Figure 1. Bars indicate mean of the waist-to-hip ratio of patients in each group. Error bars indicate standard deviation. *∗* indicates a significant difference in the measurement at 8 weeks compared with the baseline (*p* < 0.05, paired *t*-test).

**Figure 4:**
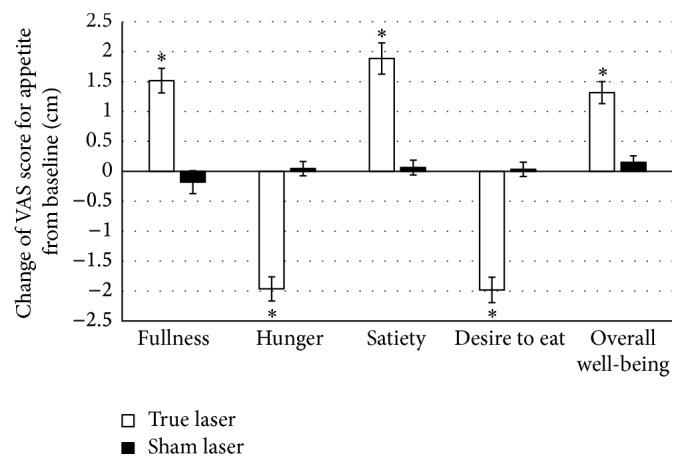
Mean changes in VAS score for fullness, hunger, satiety, desire to eat, and overall well-being after true LA and sham LA treatment (*n* = 52). Changes are calculated as the difference between the value at 8 weeks and the baseline value for each patient. Error bars represent SEM of the VAS scores. *∗* indicates a significant difference in the measurement at 8 weeks compared with the baseline (*p* < 0.05, paired *t*-test).

True LA led to significant reductions in BMI (primary outcome measurement). BMI decreased from 31.2 ± 5.3 to 30.6 ± 5.5 kg/m^2^ (*p* < 0.01) (Figure 2). The results of secondary outcome measurements including body fat and WHR were all significantly reduced in true LA group (*p* < 0.01). Body fat decreased from 40.1 ± 8.5 to 39.1 ± 8.7%. WHR decreased from 0.87 ± 0.07 to 0.84 ± 0.07 (Figure 3). In addition, the waist and hip circumferences were significantly reduced (−3.2 ± 3.4 and −0.7 ± 2.2 cm, resp., for both). All subjects that were given true LA reported that their appetite sensation improved after the trial and attributed this to the interventions that were performed. True LA significantly improved scores on the fullness, hunger, satiety, desire to eat, and overall well-being in relation to the baseline (*p* < 0.01 for all) (Figure 4). These changes are significantly different from the changes from the sham LA group (*p* < 0.01 for all variables) (Table 3).

## 4. Discussion

Through this study we discovered that LA may have favorable effects on obesity. BMI, body fat, and WHR all decreased over the course of the LA intervention. These findings are consistent with those reported by other studies [14, 15, 18]. Unlike previous studies utilizing combination interventions and inadequate placebo controls, this patient-assessor-blinded, randomized, sham-controlled crossover trial helps elucidate the substantial variability in outcomes. The crossover design has reduced between-patient variation compared to the case-control design because each participant acts as his or her own matched control [30, 31]. Moreover, laser acupuncture did not produce a noticeable sensation that would lead to order and learning effects in the subjects. A 1-week washout period between treatment sessions was used in a previous randomized crossover study of acupuncture on obesity [32]. We extended this washout period to 2 weeks to further reduce potential carryover effects. No adverse events were reported by the subjects of this clinical investigation, and none withdrew from the study. This supports the safety and feasibility of LA in the treatment of obesity.

Improvement in BMI and WHR suggests that LA may be considered a noninvasive option for body contouring with weight loss. Although weight changes over the course of the treatment would also change waist circumference, results from controlled clinical studies have demonstrated that LLLT achieved safe and significant waist circumference reduction [16, 17]. However, future investigations must be conducted to explore the long-term effects of LA on body parts for circumferential loss.

The potential underlying mechanisms of LA on obesity seem to be unclear, but the emerging view is that LA can be linked to the positive effects of acupuncture and the biological effect of LLLT. Previous studies have suggested that LA shares the principles of traditional acupuncture and has comparable biological effect [33]. The actual mechanism of LLLT on fat remains controversial. Results from preclinical and clinical studies suggest that several mechanisms may be involved, including effects on production of transitory pores in adipocytes, induction of adipocyte apoptosis, and subsequent release of lipids [34].

Another important finding from this study was that when compared to the control sessions, true LA decreased the desire to eat, decreased hunger, enhanced satiety, enhanced fullness, and enhanced well-being. To our knowledge, these secondary outcomes have not yet been measured in similar studies investigating the effects of LA on obesity. These results point towards a potential for added health benefits from LA. It is possible that the beneficial effects of LA on appetite suppression and circumferential reduction may help people achieve better self-control in diet and exercise. LA may also play a role in encouraging subjects to adhere to changes in their behavioral lifestyle.

There are some limitations to this study that should be considered. Firstly, further studies analyzing the maintaining effects of LA should be conducted. Due to the subject selection criteria, the generalizability of the results may be limited to other populations. Furthermore, given that previous research has suggested that gender moderates the psychological and behavioral variables on weight loss treatments [35], the relevance of gender on weight loss differences with respect to LA remains unexplored. A larger study with equal sample sizes between the two genders is required to clarify this substantial variability in outcomes and subsequently generalize our findings.

## 5. Conclusion

LA improves BMI, fat mass, WHR, waist circumference, and hip circumference, decreases hunger, and decreases desire to eat. Furthermore, LA enhanced satiety and well-being when compared to the control. These findings suggest that LA may be a treatment option for individuals suffering from obesity. However, a long-term multicentre study involving a large number of patients is needed to confirm the above findings and to evaluate the underlying mechanisms.

## Figures and Tables

**Table 1:** Anatomical location of the acupuncture points used in this study.

Acupuncture point	Anatomical location	Function according to TCM
ST25	Middle of the abdomen, 2 cun lateral to the umbilicus.	Regulates spleen, stomach, and intestines.
ST36	3 cun below patella, 1 cun lateral of the tibial rim.	Strengthens spleen and stomach.
ST40	At the midpoint of a line between ST35 at the lateral patella and the lateral malleolus.	Transforms phlegm and dampness.
ST44	Proximal to the web margin between the 2nd and 3rd metatarsal toes, at the junction of the red and white skin.	Clears heat from stomach, resolves damp heat.
LI4	On the dorsum of the hand, between the 1st and 2nd metacarpal bones, in the middle of the 2nd metacarpal bone on the radial side.	Expels wind and releases the exterior, tonifies qi, strengthens immunity.
LI11	With the elbow flexed, the point is on the lateral end of the transverse cubital crease.	Clears heat, cools blood, resolves dampness, expels exterior wind.
SP6	3 cun above the medial malleolus, dorsal tibial rim.	Strengthens spleen and stomach, resolves dampness.
PC6	2 cun above the transverse crease of the wrist, between the tendons of m. palmaris longus and m. flexor carpi radialis.	Regulates heart qi, calms the shen, harmonizes stomach.

**Table 2:** Baseline demographic and anthropometric characteristics of study subjects.

Characteristics	Male	Female
Number of patients	11	41
Age (years)	42.6 ± 15.1	37.8 ± 10.6
Body weight (kg)	86.2 ± 14.2	79.8 ± 15.7
Height (cm)	167.4 ± 4.6	159.8 ± 5.8
Body mass index (kg/m^2^)	30.8 ± 5.3	31.2 ± 5.4
Body fat (%)	28 ± 5.6	42.7 ± 6
Waist circumference (cm)	99.8 ± 7.7	92 ± 10.8
Hip circumference (cm)	106.7 ± 8.5	110.5 ± 11.3
Waist-to-hip ratio	0.94 ± 0.06	0.84 ± 0.06
Systolic blood pressure (mmHg)	131.8 ± 6.1	122.6 ± 9.8
Diastolic blood pressure (mmHg)	76.5 ± 3.1	75 ± 5.6

All values are mean ± SD.

**Table 3:** Effect of laser acupuncture on anthropometric measurements and appetite sensation.

Variable	True laser					Sham laser					*p* value^§^	FDR
	Before	After	Change^†^	*p* value^‡^	FDR	Before	After	Change	*p* value	FDR		
Weight (kg)	81.5 ± 15.5	79.8 ± 15.7	−1.7 ± 1.5	2.5 × 10^−11^	9.3 × 10^−11^	80 ± 16.1	80.2 ± 16	0.2 ± 0.8	0.16	0.31	1.2 × 10^−12^	6.5 × 10^−12^
Body mass index (kg/m^2^)	31.2 ± 5.3	30.6 ± 5.5	−0.7 ± 0.6	4.3 × 10^−11^	1.2 × 10^−10^	30.7 ± 5.6	30.7 ± 5.6	0.07 ± 0.33	0.13	0.31	5.2 × 10^−12^	1.5 × 10^−11^
Body fat (%)	40.1 ± 8.5	39.1 ± 8.7	−0.9 ± 1.1	4.5 × 10^−8^	4.9 × 10^−8^	39.2 ± 8.6	39.5 ± 8.7	0.3 ± 1	0.065	0.24	9.3 × 10^−7^	1.0 × 10^−6^
Waist circumference (cm)	94.5 ± 10.9	91.3 ± 10.9	−3.2 ± 3.4	8.9 × 10^−9^	1.2 × 10^−8^	91.7 ± 10.6	92.5 ± 10.9	0.8 ± 1.8	0.0027	0.015	7.8 × 10^−10^	1.2 × 10^−9^
Hip circumference (cm)	109.4 ± 10.7	108.7 ± 10.7	−0.7 ± 2.2	0.02	0.02	109.2 ± 10.8	108.9 ± 10.9	−0.3 ± 1.7	0.23	0.36	0.22	0.22
Waist-to-hip ratio	0.87 ± 0.07	0.84 ± 0.07	−0.02 ± 0.03	4.4 × 10^−8^	4.9 × 10^−8^	0.84 ± 0.07	0.85 ± 0.07	0.01 ± 0.02	1.5 × 10^−4^	0.0017	1.3 × 10^−10^	2.8 × 10^−10^
Fullness score (cm)	3.8 ± 2.1	5.3 ± 1.9	1.5 ± 1.5	1.2 × 10^−9^	2.6 × 10^−9^	4.4 ± 2.4	4.2 ± 2.1	−0.2 ± 1.4	0.35	0.48	1.8 × 10^−9^	2.4 × 10^−9^
Hunger score (cm)	6.6 ± 1.9	4.6 ± 1.8	−2 ± 1.5	3.7 × 10^−13^	4.0 × 10^−12^	5.6 ± 2.2	5.7 ± 2.1	0 ± 0.9	0.71	0.78	5.4 × 10^−12^	1.5 × 10^−11^
Satiety score (cm)	4.6 ± 2	6.5 ± 1.9	1.9 ± 1.9	2.9 × 10^−9^	4.5 × 10^−9^	4.8 ± 2	4.9 ± 1.9	0.1 ± 0.9	0.61	0.75	4.9 × 10^−10^	8.9 × 10^−10^
Desire-to-eat score (cm)	6.3 ± 2.1	4.3 ± 2.1	−2 ± 1.5	1.2 × 10^−12^	6.3 × 10^−12^	5.9 ± 2.3	6 ± 2.2	0 ± 0.9	0.79	0.79	7.5 × 10^−13^	6.5 × 10^−12^
Overall well-being score (cm)	4.9 ± 2.1	6.2 ± 2.1	1.3 ± 1.3	2.7 × 10^−9^	4.5 × 10^−9^	5.1 ± 2.1	5.2 ± 2	0.1 ± 0.8	0.17	0.31	8.1 × 10^−7^	9.9 × 10^−7^

All values are mean ± SD.

^†^Calculated by subtracting the values of baseline from values of week 8.

^‡^For comparison of values at baseline and week 8 of each intervention.

^§^For the comparison of mean changes between the true laser and sham laser interventions by paired *t*-test.

FDR, false discovery rate of *p* values adjusted by the Benjamini and Hochberg method.
